# Centrilobular emphysema combined with pulmonary fibrosis results in improved survival

**DOI:** 10.1186/1755-1536-4-6

**Published:** 2011-02-15

**Authors:** Nevins W Todd, Jean Jeudy, Sachin Lavania, Teri J Franks, Jeffrey R Galvin, Janaki Deepak, Edward J Britt, Sergei P Atamas

**Affiliations:** 1 Division of Pulmonary and Critical Care Medicine, Department of Medicine, University of Maryland School of Medicine, Baltimore, Maryland, USA; 2 VA Medical Center, Baltimore, Maryland, USA; 3 Department of Diagnostic Radiology, University of Maryland School of Medicine, Baltimore, Maryland, USA; 4 Department of Pulmonary and Mediastinal Pathology, Armed Forces Institute of Pathology, Washington, DC, USA

## Abstract

**Background:**

We hypothesized that, in patients with pulmonary fibrosis combined with emphysema, clinical characteristics and outcomes may differ from patients with pulmonary fibrosis without emphysema. We identified 102 patients who met established criteria for pulmonary fibrosis. The amount of emphysema (numerical score) and type of emphysema (centrilobular, paraseptal, or mixed) were characterized in each patient. Clinical characteristics, pulmonary function tests and patient survival were analysed.

**Results:**

Based on the numerical emphysema score, patients were classified into those having no emphysema (*n *= 48), trivial emphysema (*n *= 26) or advanced emphysema (*n *= 28). Patients with advanced emphysema had a significantly higher amount of smoking in pack/years than patients with no emphysema or trivial emphysema (*P *< 0.0001). Median survival [1st, 3rd quartiles] of patients with advanced emphysema was 63 [36, 82] months compared to 29 [18, 49] months in patients without emphysema and 32 [19, 48] months in patients with trivial emphysema (*P *< 0.001). Median forced vital capacity (FVC) and total lung capacity (TLC) were higher in the advanced emphysema group compared to patients with no emphysema (*P *< 0.01 and *P *< 0.001, respectively), whereas median DL_CO _did not differ among groups and was overall low. Within the advanced emphysema group (*n *= 28), further characterization of the type of emphysema was performed and, within these subgroups of patients, survival was 75 [58, 85] months for patients with centrilobular emphysema, 75 [48, 85] months for patients with mixed centrilobular/paraseptal emphysema, and 24 [22, 35] months for patients with paraseptal emphysema (*P *< 0.01). Patients with advanced paraseptal emphysema had similar survival times to patients without emphysema.

**Conclusions:**

Patients with pulmonary fibrosis combined with advanced centrilobular or mixed emphysema have an improved survival compared with patients with pulmonary fibrosis without emphysema, with trivial emphysema or with advanced paraseptal emphysema.

## Background

Pulmonary fibrosis is a major component of various diffuse parenchymal lung diseases, including idiopathic pulmonary fibrosis (IPF) and other forms of idiopathic interstitial pneumonia. Pulmonary fibrosis results in substantial morbidity and mortality and therapies have been uniformly poorly effective [1,2].

Histological changes in the lungs of patients with IPF show usual interstitial pneumonia (UIP), a pattern of fibrosis characterized by fibroblastic foci and excessive deposition of extracellular matrix. Initial hypotheses for the mechanism of IPF focused on the role of inflammation and most authorities believed that pulmonary inflammation was a prominent and necessary feature of the UIP process [3-5]. More recently, however, the role of inflammation has been questioned and, although the precise aetiology of IPF remains unknown, most experts presently state that active cellular lung inflammation is not a major feature or requirement for the development of UIP [4]. Furthermore, in addition to inflammation being considered nonessential for the development of IPF/UIP, a hypothesis was proposed that inflammation may actually be beneficial in this disease process [5]. Observations in human patients and animals provide data in support of this hypothesis. Patients with connective tissue diseases such as systemic sclerosis develop pulmonary fibrosis but this occurs in association with autoimmune inflammation and the survival of patients with interstitial pneumonia associated with connective tissue diseases is longer than patients with IPF [6,7]. We previously reported that lymphocytic inflammation induced by gene delivery of chemokine CCL18 is partially protective against the severe fibrosis caused by administration of bleomycin in mice [8]. Based on these considerations, we hypothesized that patients with IPF combined with a chronic inflammatory process may be partially protected from the usual rapidly declining clinical course. One example of a well-established chronic inflammatory pulmonary disease is emphysema [9-11]. Emphysema is most commonly caused by cigarette smoking [9] and the inflammatory component of emphysema may be only partially reversed after smoking cessation [11].

We specifically hypothesized that patients with combined pulmonary fibrosis and emphysema may have different clinical characteristics and outcomes than patients with pulmonary fibrosis in the absence of emphysema. In this study, we determined the presence or absence of emphysema, along with its severity and type, in a cohort of patients with pulmonary fibrosis and characterized their clinical characteristics, pulmonary physiology and overall outcomes.

## Methods

### Patients

The study was reviewed and approved by the University of Maryland Institutional Review Board (Human Research Protection Office, Protocol HP-00044077). Patients with interstitial pneumonia were identified at the University of Maryland either through direct patient care visits or through ICD-9 code review for 'interstitial lung disease'. Following our review, 102 patients were identified who met either (1) established criteria for definite or confident idiopathic pulmonary fibrosis (IPF) or (2) established clinical and pathologic criteria for idiopathic fibrotic NSIP (fibrotic non-specific interstitial pneumonia) [1,2]. Patients with following diagnoses were not included in this cohort: (a) other forms of idiopathic interstitial pneumonia (cryptogenic organizing pneumonia, respiratory bronchiolitis-interstitial lung disease, acute interstitial pneumonia and desquamative interstitial pneumonia); (b) connective tissue disease-interstitial pneumonia; (c) sarcoidosis; (d) hypersensitivity pneumonitis; and (e) pneumoconiosis.

Computed tomography (CT) of the chest, patient demographics and clinical characteristics, lung pathology, pulmonary function tests (PFTs), pulmonary vascular haemodynamics and overall outcomes were reviewed and recorded for each patient. The date of diagnosis was identified for each patient, defined as the date on which clinical evaluation for respiratory symptoms occurred and radiographic evidence of pulmonary fibrosis was present. Survival was calculated from the date of diagnosis to the date of death or the date of a lung transplantation, whichever came first. If neither occurred, survival was defined from the date of diagnosis to the date of review.

### Computed tomography of the chest

All patients had a CT of the chest performed and each CT was reviewed by two thoracic radiologists (JJ, JRG) specifically for the purpose of this study.

All patients had radiographic findings of pulmonary fibrosis on CT, as evidenced by a combination of reticular opacities, traction bronchiectasis, architectural distortion, honeycomb change and a peripheral and basilar predominance to these findings [1,2]. Areas of ground glass opacities were minor findings if present. The amount of fibrosis was visually assessed and quantified on CT as involving < 10%, 10%-40% or > 40% of the lung parenchyma [12].

The presence of emphysema was visually assessed in each patient according to a modification of the National Emphysema Treatment Trial (NETT) scoring system [13]. The right and left lungs were divided into an upper portion (apex to aortic arch), a mid portion (aortic arch to inferior pulmonary vein) and a lower portion (inferior pulmonary vein to diaphragm) and a score was assigned to describe the amount of lung affected by emphysema in each portion as follows: score 0 (no emphysema); score 0.5 (trivial, < 5%); score 1 (mild, 5%-25%); score 2 (moderate, 26%-50%); score 3 (marked, 51%-75%); and score 4 (severe, > 75%). The scores for each portion of the right and left lungs (six portions) were added to obtain the result of a total emphysema score, with 24 being the maximum possible score.

Additionally, the type of emphysema present was visually assessed and determined as previously described [14]. The following patterns were identified: (a) exclusively centrilobular; (b) exclusively paraseptal; (c) centrilobular-predominant, in which at least 80% of emphysema present was in centrilobular pattern; (d) paraseptal-predominant, in which at least 80% of emphysema present was in a paraseptal pattern; and (e) mixed emphysema, in which an equal amount of centrilobular and paraseptal emphysema were present.

### PFT

All PFT data available was recorded for each patient, including forced expiratory volume in one second (FEV_1_), forced vital capacity (FVC), FEV_1_/FVC ratio, total lung capacity (TLC), functional residual capacity (FRC), residual volume (RV) and diffusing capacity for carbon monoxide (DL_CO_). Most patients had multiple sets of PFT data available for review. For the purpose of this study, the set of PFT data closest to the date of the CT was used for analysis.

### Pathology

A surgical lung biopsy was obtained in 73 of the 102 patients and was defined as a lung biopsy obtained from either video assisted thoracoscopy (VATS), an explanted native lung obtained at the time of lung transplantation, or an autopsy specimen. The pathology of each patient was reviewed, and was interpreted according to the consensus statement on idiopathic interstitial pneumonia (IIP) [2].

### Right heart catheterization (RHC)

RHC was performed in 76 of the 102 patients; it was performed at the discretion of the treating physician and was usually performed as part of an evaluation for lung transplantation. Data measured at the time of RHC included systolic and diastolic pulmonary artery pressure, pulmonary capillary wedge pressure, cardiac output and mixed venous oxygen saturation. Mean pulmonary artery pressure (mPAP) was calculated as the diastolic pressure plus one-third of the pulse pressure. Pulmonary vascular resistance was calculated as mPAP minus wedge pressure, divided by the cardiac output.

### Statistical analyses

Data were analysed using Graph Pad Software (CA, USA). Differences between groups were analysed by Kruskal-Wallis one way ANOVA with Dunn's multiple comparison test or by chi-square test as indicated. Differences at *P *< 0.05 were considered significant.

## Results

### Overall cohort

The clinical characteristics of the overall cohort of 102 patients with pulmonary fibrosis are shown in Table 1. There were more males than females and more Caucasian patients than those of other racial groups. Most patients were on one or more immunosuppressive medications, with prednisone being the most commonly used. Approximately two-thirds of patients (*n *= 71) had a history of smoking and the remainder were lifelong non-smokers. RHC was performed in 76 patients and pulmonary hypertension (defined as mPAP > 25 mm Hg) was present in 41% of those patients. A surgical lung biopsy documenting histologically the presence of pulmonary fibrosis was obtained in the majority of patients. PFTs indicated a moderately severe restrictive ventilatory defect and a severe reduction in the DL_CO _for the group as a whole.

**Table 1:** Characteristics of the overall patient cohort (*n *= 102).

Age, years, *median [1*^*st*^*, 3*^*rd*^*]*	61 [55, 66]
Gender:	
Male, *n*	66
Female, *n*	36
Race:	
Caucasian, *n*	69
African-American, *n*	28
Other, *n*	5
Medication:	
Prednisone, *n*	88
Azathioprine, *n*	49
Mycophenolate, *n*	27
N-Acetylcysteine, *n*	42
ACE Inhibitor, *n*	19
Statin, *n*	45
Smoking history:	
Never, *n *(%)	31 (30)
Ever, *n *(%)	71 (70)
Right heart catheterization:	
Performed, *n *(%)	76 (75)
PH present, *n* (%)	31 (41)
Surgical lung biopsy:	
Performed, *n *(%)	73 (71)
UIP, *n*	60
Fibrotic NSIP, *n*	13
Pulmonary function tests:	
FVC [% predicted; *median (1st, 3rd)*]	54 [42, 66]
TLC [% predicted; *median (1st, 3rd)*]	54 [42, 64]
DL_CO _[% predicted; *median (1st, 3rd)*]	28 [20, 36]

PH, pulmonary hypertension (defined by mean pulmonary artery pressure > 25 mm Hg); UIP, usual interstitial pneumonia; NSIP, non-specific interstitial pneumonia; FVC, forced vital capacity; TLC, total lung capacity; DL_CO_, diffusing capacity for carbon monoxide.

### Emphysema score and survival

**Figure 1:**
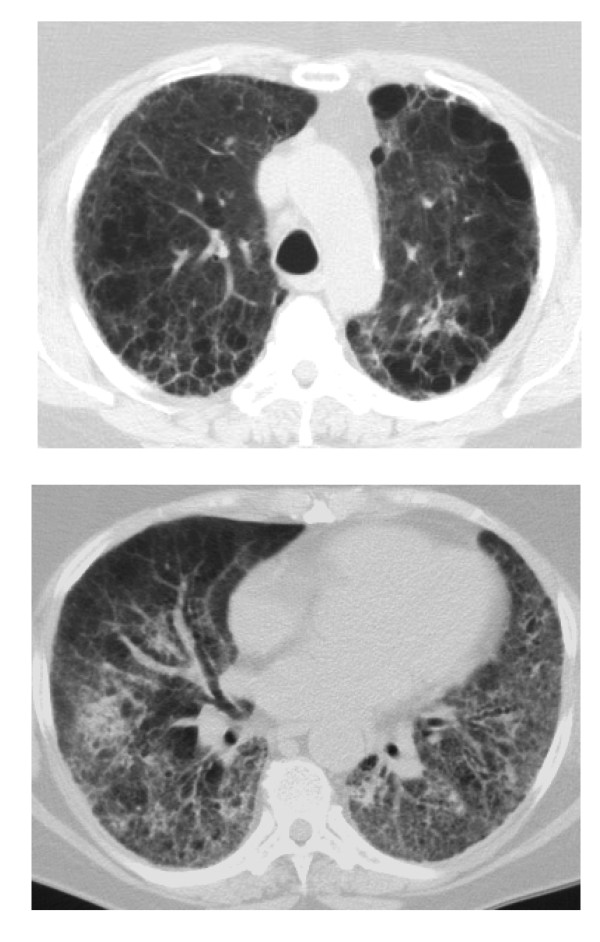
**Chest computed tomogram from a patient with pulmonary fibrosis and an emphysema score of 12**. The upper panel shows advanced predominantly centrilobular emphysema at the level of the aortic arch. The lower panel shows mild ground glass opacities, substantial reticular opacities, and advanced traction bronchiectasis at the lung bases. Surgical lung biopsy in this patient showed usual interstitial pneumonia (UIP).

The total emphysema score was calculated for each patient based on the chest computed tomography (CT). Total emphysema scores ranged from 0 to 22 in this cohort. The patients were placed into one of three groups based on the total emphysema score: (a) an emphysema score of zero (ES 0), indicating no emphysema could be detected (*n *= 48); (b) an emphysema score greater than zero but less than or equal to 2 (0 < ES ≤ 2), consistent with trivial emphysema (*n *= 26); and (c) an emphysema score greater than two (ES > 2), consistent with advanced emphysema (*n *= 28). A chest CT from a patient with ES > 2 is shown in Figure 1.

Table 2 shows the clinical characteristics of these three patient groups. There were no differences among the groups in regards to age, gender, race or medication use. More patients in the ES > 2 and 0 < ES ≤ 2 groups had a history of 'ever-smoking' but there were very few current smokers in any group. Patients with ES > 2 had a significantly higher amount of smoking in pack/years than patients with ES 0 and 0 < ES ≤ 2 (P < 0.001 and P < 0.05, respectively, Dunn's multiple comparison test), and patients with 0 < ES ≤ 2 had smoked more than patients with ES 0 (*P *< 0.05). All patients had CT findings of pulmonary fibrosis and there were no differences in the fibrosis scores between the groups. Similar proportions of patients in each group had RHC performed but patients with ES >2 and 0 < ES ≤ 2 had a higher median mPAP than patients with ES 0 (*P *< 0.05 and *P *< 0.01, respectively). Similar proportions of patients in each group had undergone a surgical lung biopsy and, although there was a trend towards more fibrotic NSIP in the ES > 2 group, this was not statistically significant (*P *> 0.05). A similar proportion of patients in each group underwent lung transplantation.

**Table 2:** Clinical characteristics of patients based on emphysema score.

Variable	ES 0 (*n *= 48)	0 < ES ≤ 2 (*n *= 26)	ES > 2 (*n *= 28)	*P *value
Age years; *median [1st, 3rd]*	62 [58, 68]	61 [53, 67]	57 [51, 62]	NS*
Gender:				
Male, *n * Female,* n*	31 17	18 8	17 11	NS^†^
Race:				
Caucasian, *n*	38	15	16	
African-American, *n*	8	9	11	NS^†^
Other, *n*	2	2	1	
Medication:				
Prednisone, *n*	40	26	22	NS^†^
Azathioprine, *n*	24	13	12	NS^†^
Mycophenolate, *n*	14	9	4	NS^†^
N-Acetylcysteine, *n*	22	13	7	NS^†^
ACE Inhibitor, *n*	10	6	3	NS^†^
Statin, *n*	20	14	11	NS^†^
Smoking:				
Current smoker, *n *(%)	1(2)	2 (7)	4 (14)	NS^†^
Ever-smoker, *n *(%)	27 (56)	21 (81)	24 (86)	< 0.01^†^
Pack/years, *median [1st, 3rd]*	5 [0, 20]	20 [10, 39]	40 [20, 50]	< 0.0001*
Fibrosis score:				
< 10%, *n*	7	5	2	
10 - 40%, *n*	27	16	19	NS^†^
> 40%, *n*	14	5	7	
Right heart catheterization:				
Performed, *n *(%)	37 (77)	22 (84)	17 (61)	NS^†^
Mean PAP mm Hg; *median [1st, 3rd]*	21 [18, 24]	29 [22, 40]	29 [22, 33]	< 0.001*
Surgical lung biopsy:				
Performed, *n *(%)	36 (75)	17 (65)	20 (71)	NS^†^
UIP, *n*	32	14	14	NS^†^
Fibrotic NSIP, *n*	4	3	6	
Lung transplantation performed, *n *(%)	19 (39)	6 (23)	9 (32)	NS^†^

*Kruskal-Wallis one-way ANOVA

^†^Chi-square test

ES, emphysema score; mean PAP, mean pulmonary artery pressure; UIP, usual interstitial pneumonia; NSIP, non-specific interstitial pneumonia; NS, not significant (*P *> 0.05).

**Figure 2:**
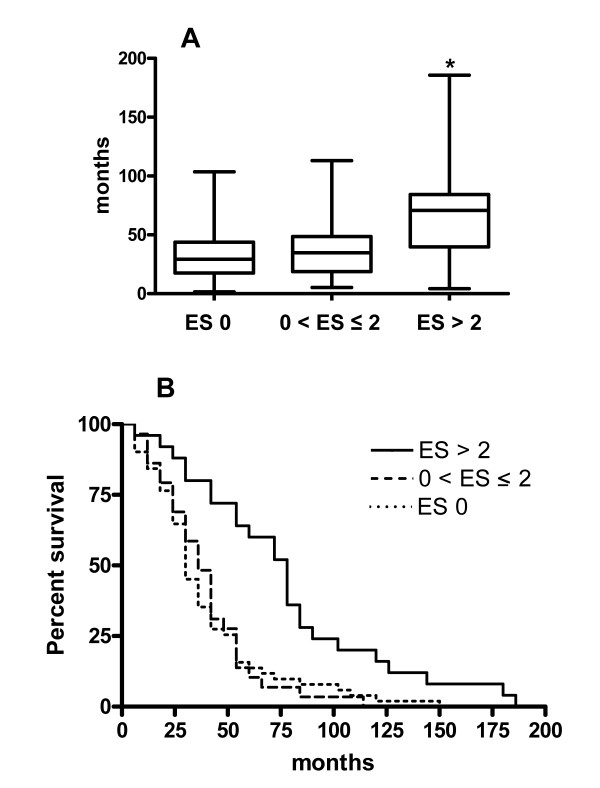
**Differences in survival based on emphysema score [ES]**. Median survival differed among the three groups (*P *< 0.001, Kruskal-Wallis one-way ANOVA). By Dunn's multiple comparison tests, patients with ES > 2 (*n *= 28) had a longer survival than patients with ES 0 (*n *= 48) and patients with 0 < ES ≤ 2 (*n *= 26) (*P *< 0.001 and *P *< 0.01, respectively). (B) Kaplan-Meier survival analyses revealed significantly longer survival in patients with ES > 2 (*P *= 0.001; log-rank test).

Survival was determined based on the status of each patient as alive, died or had undergone lung transplantation. Survival for the three groups of patients is shown in Figure 2. Median survival [1st, 3rd quartiles] in patients with ES > 2 was 63 [36, 82] months compared with 29 [18, 49] months in patients having ES 0 and 32 [19, 48] months in patients with 0 < ES ≤ 2 (*P *< 0.001). There was no difference in survival between the ES 0 group and the 0 < ES ≤ 2 group. Kaplan-Meier survival curves of the three groups of patients revealed a significantly longer survival for the ES > 2 patients (*P *= 0.001).

### PFTs

PFTs from patients in each of the three patient groups are shown in Table 3. FVC differed among the three groups and was higher in the ES > 2 group compared with the ES 0 group (*P *< 0.01, Dunn's multiple comparison test). TLC also differed among the three groups and was higher in the ES > 2 group compared with the 0 <ES ≤ 2 and ES 0 groups (*P *< 0.05 and *P *< 0.001, respectively). However, although the FVC and TLC were higher in the ES > 2 group, the percent predicted values still remained low and were consistent with an overall restrictive ventilatory defect. The FEV_1_/FVC ratio differed among the three groups and was lower in the ES > 2 compared to ES 0 group (*P *< 0.05). DL_CO _was low in all groups and did not differ among groups.

**Table 3:** Pulmonary function tests (PFTs)

**Variable**	**ES 0** **(*n *= 48)**	**0 < ES ≤ 2** **(*n *= 26)**	**ES > 2** **(n = 28)**	*P value
	
FEV_1 _% predicted; *median [1st, 3rd]*	53 [43, 74]	59 [48, 77]	66 [60, 76]	NS
FVC % predicted; *median [1st, 3rd]*	47 [38, 63]	50 [43, 62]	60 [55, 73]	< 0.01
FEV_1_/FVC ratio *median [1st, 3rd]*	84 [81, 88]	87 [80, 89]	81 [72, 86]	< 0.05
TLC % predicted; *median [1st, 3*^*rd*^*]*	44 [39, 57]	50 [44, 59]	64 [57, 73]	<.001
DL_CO_, % predicted; *median [1st, 3rd]*	28 [19, 34]	31 [20, 36]	27 [20, 37]	NS

*Kruskal-Wallis one-way ANOVA ES, emphysema score; FEV_1_, forced expiratory volume in one second; FVC, forced vital capacity; TLC, total lung capacity; DL_CO_, diffusing capacity for carbon monoxide; NS, not significant (*P *> 0.05).

### Emphysema type and survival in the advanced emphysema (ES > 2) group

As patients with ES >2 (*n *= 28) demonstrated a prolonged survival, we further characterized the type of emphysema present in each patient within this advanced emphysema group. Patients were placed into three groups based on the following characterizations: (a) paraseptal emphysema (*n *= 7), which included patients with exclusively paraseptal emphysema and paraseptal-predominant emphysema; (b) centrilobular emphysema (*n *= 13), which included patients with exclusively centrilobular emphysema and centrilobular-predominant emphysema; or (c) mixed emphysema (*n *= 8).

The clinical characteristics of these three subgroups of patients are shown in Table 4. There were no differences among the groups with regards to age or race but there were more females than males in the centrilobular group. Patients with centrilobular or mixed emphysema had a history of higher amounts of smoking (*P *< 0.05). A UIP pattern of fibrosis was seen in all subgroups, although there was a higher proportion of fibrotic NSIP in the centrilobular group. TLC differed among the groups but there were no differences in DL_CO_.

**Table 4:** Clinical characteristics of patients with emphysema score (ES) > 2 based on type of emphysema.

Variable	ES > 2 Paraseptal (*n *= 7)	ES > 2 Mixed (*n *= 8)	ES > 2 Centrilobular (*n *= 13)	*P *value
Age years; *median [1st, 3rd]*	57 [51, 63]	59 [57, 63]	54 [49, 62]	NS*
Gender:				
Male, *n*	7	8	2	< 0.001^†^
Female, *n*	0	0	11	
Race:				
Caucasian, *n*	3	6	7	
African-American, *n*	3	2	6	NS^†^
Other, *n*	1	0	0	
Smoking packs/years; *median [1st, 3rd]*	14 [0, 38]	40 [38, 46]	45 [25, 60]	< 0.05*
Right heart catheterization:				
Performed, *n *(%)	5 (71)	6 (75)	6 (46)	NS^†^
Mean PAP mm Hg; *median [1st, 3rd]*	32 [21, 34]	31 [29, 33]	25 [22, 31]	NS*
Surgical lung biopsy:				
Performed, *n *(%)	6 (86)	5 (63)	9 (70)	NS^†^
UIP, *n*	6	4	4	< 0.05^†^
Fibrotic NSIP, *n*	0	1	5	
FVC % predicted; *median, [1st, 3rd]*	57 [50, 60]	59 [51, 63]	71 [58, 84]	NS*
TLC % predicted; *median, [1st, 3rd]*	58 [51, 64]	60 [52, 69]	72 [64, 86]	0.05
DL_CO _% predicted; *median, [1st, 3rd]*	25 [21, 31]	28 [23, 34]	27 [18, 37]	NS*

*Kruskal-Wallis one-way ANOVA; ^†^Chi-square test.

Paraseptal, paraseptal emphysema; mixed, mixed centrilobular and paraseptal emphysema; centrilobular, centrilobular emphysema; mean PAP, mean pulmonary artery pressure; UIP, usual interstitial pneumonia; NSIP, non-specific interstitial pneumonia; FVC, forced vital capacity; TLC, total lung capacity; DL_CO_, diffusing capacity for carbon monoxide; NS, not significant (*P *> 0.05).

**Figure 3:**
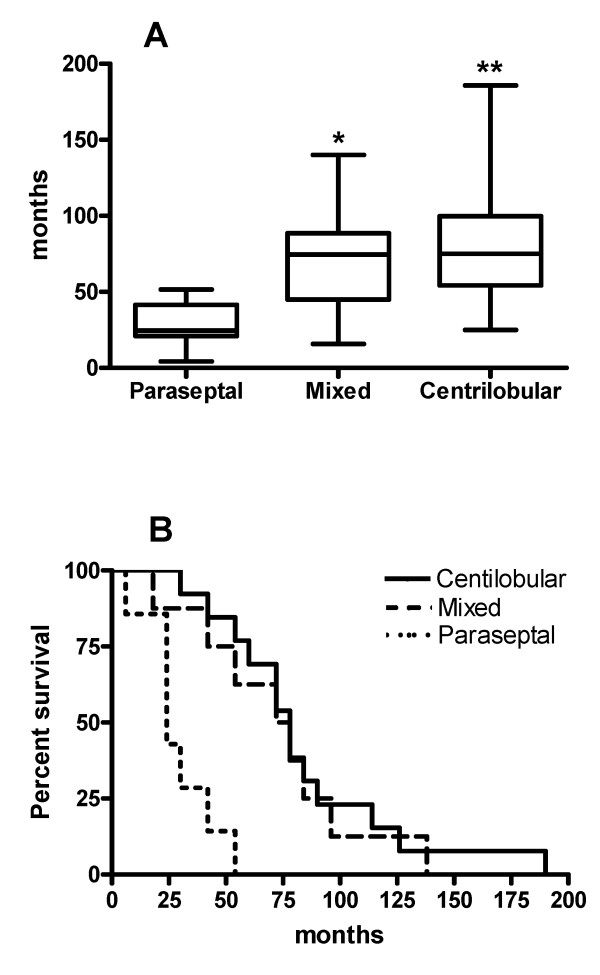
**Differences in survival among subgroups of patients with advanced emphysema (ES > 2) based on emphysema type**. (A) Median survival differed among the three groups (*P *< 0.01; Kruskal-Wallis one-way ANOVA). By Dunn's multiple comparison tests, patients with centrilobular emphysema (*n *= 13) or mixed emphysema (*n *= 8) had a longer survival than patients with paraseptal emphysema (*n *= 7)(*P *< 0.01 and *P *< 0.05, respectively). There was no difference in survival between the centrilobular group and the mixed emphysema group. (B) Kaplan-Meier survival curves of these subgroups of patients revealed a significantly longer survival for the centrilobular and mixed emphysema patients (*P *< 0.001, log-rank test).

The median survival [1st, 3rd quartiles] of these three subgroups is shown in Figure 3 and differed among the subgroups: 75 [58, 85] months in patients with centrilobular emphysema, 75 [48, 85] months in patients with mixed emphysema and 24 [22, 35] months in patients with paraseptal emphysema (*P *< 0.01). Kaplan-Meier survival curves of these subgroups of patients revealed a significantly longer survival for the centrilobular and mixed emphysema patients (*P *< 0.001).

## Discussion

We addressed the hypothesis that the prognosis of patients with pulmonary fibrosis combined with emphysema may be different from patients with pulmonary fibrosis in the absence of emphysema. We found that patients with pulmonary fibrosis and advanced emphysema (a modified NETT emphysema score > 2) survived longer than patients with fibrosis without emphysema and, furthermore, the prolonged survival benefit in patients with advanced emphysema was seen in patients with a substantial centrilobular component to their emphysema (those with centrilobular emphysema or mixed emphysema). Patients with fibrosis and trivial emphysema, as well as patients with advanced paraseptal emphysema, had survival times similar to those of patients with fibrosis and no emphysema.

Other groups have previously studied patients with a combination of emphysema and fibrosis [15-18] and, similar to our observations, they have reported emphysema located predominantly in the upper lobes, pulmonary fibrosis in the lower lobes and moderate to severe reductions in the diffusing capacity for carbon monoxide (DL_CO_) [15,16]. Reported survival rates for patients with fibrosis and emphysema have been conflicting and have been reported to be worse, unchanged or better than for patients with IPF [16-18]. We expanded the observations of these previous studies by numerically scoring emphysema in all patients, by adding matching control groups and by analysing the type of emphysema present. The novelty of our findings, based on our quantitative approach, is the improved survival in patients with fibrosis who concurrently had advanced centrilobular emphysema.

Centrilobular emphysema is almost uniformly caused by cigarette smoking [9,14] and, as expected, our patients with advanced emphysema had a substantially higher amount of past smoking history. Both cigarette smoking and centrilobular emphysema are associated with pulmonary inflammation [9] and the pro-inflammatory cytokines seen with cigarette smoking and with emphysema are also antifibrotic [19]. Paraseptal (or subpleural) emphysema is also associated with smoking but, additionally, may occur in non-smokers, is most often found in younger patients and probably has a different pathogenesis than centrilobular emphysema [14,20]. In support of these concepts, eight patients in our overall cohort who were lifelong non-smokers had radiographic findings of paraseptal emphysema. It may be reasonable to conclude that there may be less pulmonary inflammation present in patients with paraseptal emphysema versus centrilobular. The improved prognosis seen in our patients with advanced centrilobular emphysema would be consistent with our hypothesis that a chronic inflammatory process may be partially protective against the adverse effects of pulmonary fibrosis.

The mechanistic relationship between the development of emphysema and the development of fibrosis in patients with both processes remains unknown. It may be that this relationship is merely one of coincidence; patients with a history of smoking develop emphysema and then subsequently develop fibrosis unrelated to the presence of emphysema [21]. Alternatively, heavy cigarette smoking may be the common underlying cause for emphysema and fibrosis in patients with both processes, as cigarette smoke has been shown to cause fibrosis in both humans and animals [22,23]. In either instance, the presence of emphysema will be associated with pulmonary inflammation. Although it may seem paradoxical that advanced emphysema with fibrosis would improve survival, our data support the hypothesis that pulmonary inflammation may be partially protective.

The patients in our study who had advanced emphysema did have higher lung volumes (vital capacity and TLC) than patients without emphysema, a finding not surprising due to the presence of emphysema and a finding which had been previously reported in patients with emphysema and fibrosis [16]. One could argue that these higher lung volumes may contribute to the improved survival, although the DL_CO_, an indicator of abnormal gas exchange that is linked to mortality in pulmonary fibrosis, was very low in all groups [24]. The FEV_1_/FVC ratio was also lower in the advanced emphysema group but the overall physiology pattern of PFTs was still consistent with a restrictive ventilatory defect, a pattern seen in pulmonary fibrosis. This restrictive PFT pattern indicates that, despite the presence of advanced emphysema in this group, the effects of the fibrotic component dominate from a physiologic standpoint.

An issue which warrants discussion is our inclusion of patients with fibrotic NSIP. We believe inclusion of these patients was appropriate for several reasons which include: the occurrence in many instances of both NSIP and UIP patterns in different lobes from the same patient [25]; the fact that the majority of patients with UIP on biopsy and explant specimens will have NSIP areas [26]; and the known observation that smoking may cause an NSIP pattern of fibrosis [27]. Additionally, patients with UIP or NSIP but a DL_CO _< 35% predicted (median DL_CO _in our overall cohort was 28% predicted) had similar outcomes irrespective of their pathologic pattern [28].

A limitation to our study is its observational and retrospective design. Another limitation is that we did not measure the levels of pulmonary or systemic pro- or anti-inflammatory or pro- or anti-fibrotic cytokines. This limitation will be addressed in our future studies.

In summary, we have demonstrated that patients with pulmonary fibrosis and advanced emphysema (based on a modified NETT emphysema score of greater than 2) have a better prognosis than patients with pulmonary fibrosis without emphysema or with trivial amounts of emphysema. Furthermore, this improved prognosis was limited to patients with a substantial centrilobular component to their emphysema, as patients with isolated advanced paraseptal emphysema had outcomes similar to patients without emphysema. The precise mechanisms responsible for this improved survival are unknown but our data support the hypothesis that pulmonary inflammation resulting from the presence of emphysema may be partially protective against the deleterious effects of pulmonary fibrosis.

## Conclusions

We have demonstrated that patients with pulmonary fibrosis and advanced centrilobular emphysema, a well established chronic inflammatory condition of the lung, have a better prognosis than patients with pulmonary fibrosis without emphysema or those with fibrosis and trivial amounts of emphysema. Although the precise mechanisms responsible for this improved survival are not known, our data support the hypothesis that pulmonary inflammation may be partially protective against the usual deteriorating clinical course of patients with idiopathic pulmonary fibrosis.

## Abbreviations

DL _CO_: diffusing capacity for carbon monoxide; ES: emphysema score; FEV: forced expiratory volume; FVC: forced vital capacity; IPF: idiopathic pulmonary fibrosis; mPAP: mean pulmonary artery pressure; NSIP: non-specific interstitial pneumonia; PFT: pulmonary function test; RHC: right heart catheterization; TLC: total lung capacity; UIP: usual interstitial pneumonia.

## Competing interests

The authors declare that they have no competing interests.

## Authors' contributions

This manuscript has been seen and approved by all authors and all authors contributed sufficiently to warrant their inclusion in the author list and to take responsibility for the content of this report. The study was designed by NWT and SPA. NWT had full access to the data and vouches for the integrity of the data analysis. Clinical data were collected and analysed by NWT, SL, JD and EJB. Pathology materials were reviewed and analysed by NWT and TJF. Radiology data was reviewed and analysed by JJ, JRG and NWT. The database was maintained by NWT and SPA, who also performed statistical analyses and prepared the manuscript.
